# Syncytial Variant of Nodular Sclerosis Classical Hodgkin’s Lymphoma With Hemophagocytic Lymphohistiocytosis: A Rare and Under-Characterized Entity

**DOI:** 10.7759/cureus.110361

**Published:** 2026-06-06

**Authors:** Zoya Hasan, Muhammed Yaseen O P, Prerna Arora, Shramana Mandal, Sunita Aggarwal

**Affiliations:** 1 Pathology, Maulana Azad Medical College, Delhi, IND; 2 Internal Medicine, Maulana Azad Medical College, Delhi, IND

**Keywords:** abvd regime, classical hodgkin’s lymphoma, hemophagocytic lympho-histiocytosis, hodgkin and reed-sternberg (hrs) cells, immunohistochemistry, nodular sclerosis classical hodgkin’s lymphoma, nschl, prognosis, syncytial variant

## Abstract

Syncytial variant of nodular sclerosis classical Hodgkin’s lymphoma (NSCHL) is a rare morphological subtype of classical Hodgkin’s lymphoma (CHL). The Hodgkin and Reed-Sternberg (HRS) cells in syncytial variant NSCHL form confluent sheets. Few studies have reported the development of concurrent hemophagocytic lympho-histiocytosis (HLH) with Hodgkin’s lymphoma; however, no case of this variant with HLH has been reported before. We present a case of a rare variant of NSCHL with secondary HLH in a 15-year-old male patient presenting with B symptoms of high-grade fever, weight loss, and left-sided cervical swelling. Hemogram revealed pancytopenia. Biochemical investigations were suggestive of HLH. Lymph node biopsy had areas of sclerosis, nodularity with atypical mitosis, and clusters of HRS cells. The bone marrow examination showed an increase in plasma cells and histiocytes, with hemophagocytosis, along with the presence of HRS cells and epithelioid cell granulomas in a reactive background comprising plasma cells and eosinophils. Immunohistochemistry was suggestive of Hodgkin’s lymphoma, and a final diagnosis of syncytial variant of NSCHL with secondary HLH was made. Patients frequently exhibit B symptoms and are often diagnosed at a more advanced stage of disease.

## Introduction

Classical Hodgkin’s lymphoma (CHL) is a neoplasm derived from germinal centre B-cells characterized by a low fraction of tumor cells embedded in a reactive microenvironment rich in immune cells. The incidence of CHL varies, with an incidence of 2.63 per 100,000 person-years reported in the United States Surveillance, Epidemiology and End Results (SEER) data. A higher incidence is observed in high-income countries, while a higher mortality is found in low-income countries. The current classification recognizes four subtypes of CHL: Nodular Sclerosis (NSCHL), Mixed Cellularity (MCCHL), Lymphocyte-rich (LRCHL), and Lymphocyte-depleted (LDCHL). The most common subtype is NSCHL, followed by MCCHL, accounting for 58.1% and 18.9% of CHL cases, respectively. Male patients have a higher risk than female patients, except for NSCHL, which has a higher incidence in females. CHL shows a bimodal age distribution in White and Asian populations, while MCCHL is more commonly seen in children with a gradual increase in incidence with age [1,2].

The syncytial variant of NSCHL is a rare morphological subtype with an extremely rare incidence and thus has received little attention in the literature. Though first described by Lukes and Butler over 40 years ago, very few studies have attempted to define the morphological characteristics of this variant [3,4]. In the syncytial variant of NSCHL, Hodgkin/Reed-Sternberg (HRS) cells tend to cluster closely together, creating broad cohesive sheets of tumor cells. The surrounding background commonly contains areas of necrosis along with numerous granulocytic inflammatory cells. This subtype is most often seen in young adults, who typically present with lymph node enlargement, particularly in the cervical region, and may also have a mediastinal mass. Patients frequently exhibit B symptoms and are often diagnosed at a more advanced stage of disease. Large number of neoplastic cells presents a diagnostic challenge, with differential diagnoses include non-Hodgkin’s lymphomas (NHLs), anaplastic large cell lymphomas (ALCL), gray zone lymphomas (GZL) and metastatic malignancies, particularly carcinomas, and melanomas [3,5].

Few studies have reported the development of concurrent hemophagocytic lympho-histiocytosis (HLH) with Hodgkin's lymphoma (HL) [6]; however, no case of this variant with HLH has been reported before. Hereby, we present a case of rare variant of NSCHL with secondary HLH, with emphasis on vigilant surveillance and timely intervention in such complex scenarios, as this variant has poorer prognosis in patients treated with traditional standard-of-care therapy for CHL, especially if not treated early.

## Case presentation

A 15-year-old Indian male patient presented to the Medicine outpatient services with complaints of high-grade fever, on and off, for seven months, along with cervical swellings and weight loss. There was no history of night sweats. The patient had a history of pulmonary tuberculosis one year back, for which he was started on anti-tubercular therapy (ATT), but stopped the therapy before completion of the course. No significant family history was present.

On physical examination, left-sided cervical lymphadenopathy was noted from level II lymph nodes (approximately 2.5 cm X 2.5 cm in size) up to level IV lymph nodes (approximately 2 cm X 1.5 cm in size) along with the presence of hepatosplenomegaly (liver span - 18 cm and spleen span - 20 cm).

The complete blood count (CBC) and biochemical parameters have been described in Table 1.

**Table 1:** Hematological and biochemical parameters

S. No.	Parameter	Results	Normal range
1.	Hemoglobin (Hb)	5.3 gm/dL	13-17 gm/dL
2.	Total Leucocyte count (TLC)	1710/mm^3^	4000-11000/ mm^3^
3.	Absolute Neutrophil Count (ANC)	1248/mm^3^	2000-7000/mm^3^
4.	Platelet Count	26000/mm^3^	150000-410000/mm^3^
5.	Erythrocyte Sedimentation Rate (ESR)	145 mm/hr	=10 mm/hr
6.	Serum Ferritin	2364 ng/mL	30-300 ng/mL
7.	Serum Fibrinogen	50 mg/dL	200-400 mg/dL
8.	Serum Triglycerides	600 mg/dL	<150 mg/dL
9.	C-Reactive Protein	275.3 mg/L	<1 mg/L
10.	Viral Serology: Ebstein Barr Virus (EBV)	Negative	--
11.	HIV	Negative	--
12.	Cytomegalovirus (CMV)	Negative	--
13.	Hepatitis B	Negative	--
14.	Hepatitis C	Negative	--
15.	Blood culture	No organism detected	--
16.	Urine culture	No organism detected	--

The peripheral smear (PS) revealed pancytopenia with non-severe neutropenia. No circulating abnormal lymphoid cell or blastoid cell was seen on PS. Despite repeated blood cultures and urine cultures, there was no detectable bacterial infection.

Contrast enhanced computed tomography (CECT) abdomen revealed multiple enlarged peripancreatic lymph nodes along with multiple ill-defined hypodense lesions diffusely scattered in the entire liver and splenic parenchyma (Figure 1).

**Figure 1:**
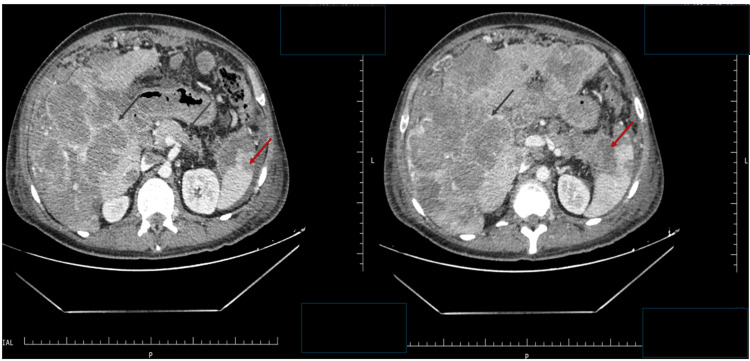
CECT abdomen Contrast enhanced computed tomography (CECT) abdomen revealed multiple enlarged peripancreatic lymph nodes (green arrow) along with multiple ill-defined hypodense lesions diffusely scattered in the entire liver (blue arrow) and splenic parenchyma (red arrow).

Based on clinical and radiological investigations, differentials of lymphoma, acute leukemia, disseminated koch’s and HLH were considered.

The bone marrow examination was done in keeping with the clinical presentation and differentials. The aspirate smears were particulate and cellular with unremarkable erythroid, myeloid and megakaryocytic series. There was an increase in plasma cells (10%) and histiocytes, with the histiocytes showing hemophagocytosis. Also noted were a few abnormal large mononuclear cells with opened up nuclear chromatin, prominent nucleoli and mild-moderate amount of cytoplasm. Bone marrow biopsy showed well-defined epithelioid cell granulomas with large areas of necrosis, granulocytic inflammation admixed with mononuclear cells with prominent eosinophilic nucleoli (Figure 2a).

**Figure 2:**
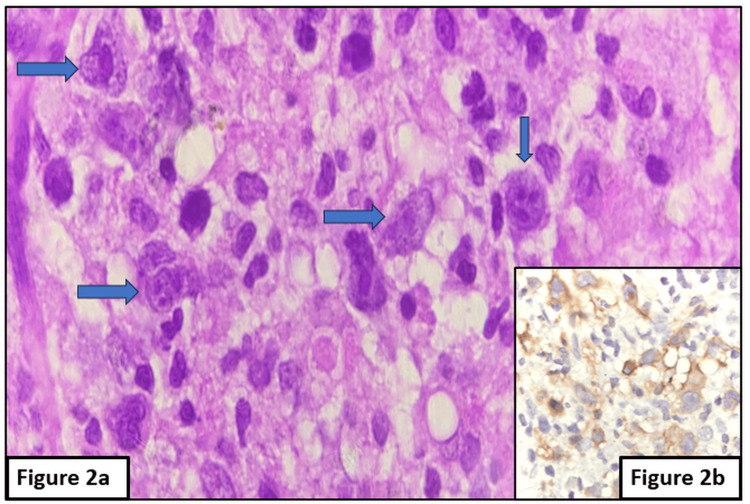
Bone marrow biopsy 2a: Clusters of Reed-Sternberg (RS) cells (mononuclear with prominent eosinophilic nucleolus) with hematoxylin and eosin (H&E) staining (1000X magnification; blue arrow); 2b: Inset (CD 30 positive RS cells with dot like and membranous staining: Immunohistochemistry with 3,3'-Diaminobenzidine (DAB) chromogen, 400x)

At places, clusters of these cells were noted. Occasional multinucleated cells were also found scattered interstitially with hematopoietic cells. There was a marked increase in histiocytes in the marrow along with presence of raised plasma cells, eosinophils and lymphocytes. On immunohistochemistry, the mononuclear cells were positive for cluster of differentiation (CD)30 (Figure 2b) and CD15, with dim positivity for paired box 5 (PAX5) and were negative for CD3, CD20, CD19, CD10 and epithelial membrane antigen (EMA) and anaplastic lymphoma kinase (ALK). Staining for acid-fast bacilli (AFB) was negative.

Excisional biopsy from left cervical lymph nodes revealed effacement of lymph node architecture with presence of mononuclear, binucleated and multinucleated lacunar cells (Reed-Sternberg or RS cells), focally in clusters, admixed with histiocytes with rare, atypical mitosis (01/20 hpf) (Figure 3a).

**Figure 3:**
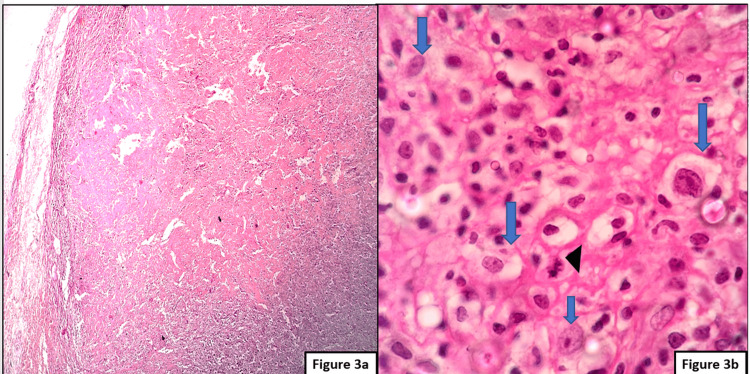
Lymph node biopsy 3a: Lymph node biopsy with areas of nodularity and sclerosis (hematoxylin and eosin staining; H&E 200X magnification); 3b: Histopathological section of Lymph node biopsy revealing syncytial sheets of lacunar Reed-Sternberg (RS) cells (arrow) along with atypical mitosis (arrow head), H&E 1000X magnification.

Areas of necrosis and ill-defined nodularity were seen, highlighted by collagen stain (Figure 3b). These cells were positive for CD15 and CD30 and negative for CD19, CD20, CD3, ALK, EMA, octamer-binding protein 2 (OCT2), B-cell lymphoma 6 (Bcl6) and CD10. Staining for AFB was negative.

A diagnosis of syncytial variant of nodular sclerosis CHL with secondary HLH with Stage IVB was made based on clinical presentation, biochemical parameters and morphological characteristics. The patient was started on HL-based therapy (ABVD regime including the drugs Adriamycin, Bleomycin, Vinblastine and Dacarbazine) and showed good response with significant clinical improvement on follow-up at six months (partial remission).

## Discussion

Syncytial variant of NSCHL is a rare variant, with few studies describing its morphological and immunophenotypic characteristics. It is a more aggressive subtype, with patients often having a higher frequency of B-type symptoms, like fever and weight loss, and advanced-stage disease. The patient in the present case also presented with high-grade fever, significant weight loss, and associated B symptoms. The immunophenotype of the syncytial variant is typical of CHL, i.e., expression of CD30, Dim PAX5, CD15 (in 65-75% of cases), and negativity for CD45/leukocyte common antigen (LCA) and other B-cell markers, such as CD19, CD22, CD79a, OCT2, and B-cell Oct-binding protein 1 (BOB1). Approximately 20% of cases show variable CD20 expression [3,5].Patients are treated similarly to typical NSCHL and have shown a good response, as in the present case [3].

HLH is a hyper-inflammatory condition that results in multi-organ failure and death. It occurs due to inappropriate and dysregulated activation of natural killer (NK) cells, CD8+ cytotoxic T-cells, and macrophages. Secondary HLH in adults occurs because of a response to an acute illness trigger rather than an underlying genetic mutation. The most common triggers include infection, malignancy, and autoimmune disorders. The development of concurrent HLH and lymphomas (both HLs and NHLs) has been reported before, but the association of syncytial variant of NSCHL and HLH has never been described before [6,7]. The original HLH-94 criteria [8] included the following features, of which all five must be met to establish a diagnosis: fever, cytopenias (at a minimal two lineages), splenomegaly, hypertriglyceridemia +/- hypofibrinogenemia, and biopsy-proven hemophagocytosis. In the HLH-2004 criteria [9], the following three additional laboratory findings were added, and the diagnosis is established by the presence of at least five of the now combined eight total criteria: serum ferritin >500 ng/ml, low or absent NK-cell activity and elevated serum IL2Ra levels ≥2400 U/mL. In the present case, six out of the eight essential diagnostic criteria’s of HLH as per HLH-2004 were met except low or absent NK-cell activity and elevated serum Interleukin 2 Receptor subunit alpha (IL2Ra) levels, with an H-Score of 195 and an 80-88% probability of HLH. Early hematopoietic stem cell transplant (HSCT) represents the only curative measure. Although adults can have a spontaneous remission of HLH, the overall mortality rate remains quite high at approximately 41% [6]. 

Diagnosis of the syncytial variant of NSCHL is difficult, since certain lymphomas can result in a similar clinical and morphological presentation. LDCHL can also show sheets of neoplastic cells mimicking syncytial variant NSHL. Histologically, lymphocytes in the background are few, and histiocytes are usually abundant in LDCHL. The well-formed collagenous bands seen in NSCHL are usually absent in lymphocyte-depleted HL (LDHL). LDHL is frequently associated with Epstein-Barr Virus (EBV). By contrast, syncytial variant NSCHL cases are usually negative for EBV, as in the present case [1,3].

The neoplastic cells in ALCL often show a broad morphological spectrum, and some ALCL cases show features that can mimic NSCHL, with tumor nodules partially surrounded by fibrous bands. There are two major types of systemic ALCL are ALK-positive, and ALK-negative. The expression of ALK does not occur in NSCHL, and therefore assessing ALK is important in such cases. A useful marker in the differential diagnosis is PAX-5. In most cases, ALK-negative ALCL shows a T-cell or null-cell phenotype, whereas NSCHL is a B-cell malignancy in which Hodgkin and Reed-Sternberg (HRS) cells typically demonstrate weak PAX-5 expression. Features favoring ALK-negative ALCL include the presence of characteristic hallmark cells with eccentric, horseshoe-shaped or reniform nuclei. Immunohistochemical expression of T-cell markers such as CD2, CD3, CD4, and CD43, along with EMA and cytotoxic proteins, further supports a diagnosis of ALK-negative ALCL [3].

GZL often contains abundant neoplastic cells with variable morphology, including large pleomorphic cells that may resemble HRS cells. These tumors most commonly arise in the mediastinum. Because many GZL cases demonstrate cohesive sheets of tumor cells, the syncytial variant of NSCHL is frequently considered in the differential diagnosis [10]. Clinically, GZL is generally associated with a less favorable prognosis compared with NSCHL [11,12]. On immunophenotyping, the tumor cells in GZL usually express CD30, CD20, CD79a, and PAX5. CD45 and Multiple Myeloma Oncogene 1/Interferon Regulatory Factor 4 (MUM1/IRF4) are also commonly positive, while CD15 expression is seen in approximately half of the cases [3].

Metastatic malignancies (e.g., carcinomas and melanomas) can also result in similar morphological presentation, however with fewer immune cells in the background. Immunohistochemistry is helpful in such cases; as most carcinomas are positive for keratins and melanomas are positive for S100, Human Melanoma Black 45 (HMB-45) and Melan-A [3].

Therefore, a critical cytomorphological assessment and immunophenotypic characteristics are helpful in diagnosis of such rare lymphomas, which can result in adverse clinical outcome if not treated early especially with co-existent HLH.

## Conclusions

The syncytial variant of NSCHL is a rare morphological subtype that can pose significant diagnostic challenges. Its clinical and histopathological features may closely resemble those of certain NHLs, GZL, and metastatic malignancies, potentially leading to diagnostic uncertainty. Careful clinicopathological correlation, supported by appropriate immunohistochemical studies, is essential for accurate diagnosis. The coexistence of secondary HLH further complicates the clinical presentation and is associated with a potentially life-threatening course. Early recognition of both the underlying lymphoma and HLH is therefore critical. Prompt initiation of appropriate therapy may improve patient outcomes and reduce disease-related morbidity and mortality.
